# Application of the FISH Technique to Visualize Sex Chromosomes in Domestic Cat Spermatozoa

**DOI:** 10.3390/ani11072106

**Published:** 2021-07-15

**Authors:** Barbara Kij-Mitka, Halina Cernohorska, Svatava Kubickova, Sylwia Prochowska, Wojciech Niżański, Joanna Kochan, Monika Bugno-Poniewierska

**Affiliations:** 1 Department of Animal Reproduction, Anatomy and Genomics, University of Agriculture, Mickiewicza 24/28, 30-059 Krakow, Poland; joanna.kochan@urk.edu.pl (J.K.); monika.bugno-poniewierska@urk.edu.pl (M.B.-P.); 2 Veterinary Research Institute, Hudcova 296/70, 621 00 Brno, Czech Republic; cernohorska@vri.cz (H.C.); kubickova@vri.cz (S.K.); 3 Department of Reproduction and Clinic of Farm Animals, Wrocław University of Environmental and Life Sciences, Grunwaldzki Square 49, 50-366 Wrocław, Poland; sylwia.prochowska@upwr.edu.pl (S.P.); wojciech.nizanski@upwr.edu.pl (W.N.)

**Keywords:** FISH, cat spermatozoa, WCPP

## Abstract

**Simple Summary:**

The study was conducted with the aim of visualizing sex chromosomes in domestic cat sperm. Our research was carried out using fluorescence in situ hybridization (FISH) technique and the microscopic analysis showed the presence of X and Y chromosomes in domestic cat spermatozoa. The procedure used for sperm decondensation and FISH is adequate to visualize chromosomes in domestic cat sperm.

**Abstract:**

Fluorescence in situ hybridization is a molecular cytogenetics technique that enables the visualization of chromosomes in cells via fluorescently labeled molecular probes specific to selected chromosomes. Despite difficulties in carrying out the FISH technique on sperm, related to the need for proper nuclear chromatin decondensation, this technique has already been used to visualize chromosomes in human, mouse, cattle, swine, horse, and dog spermatozoa. Until now, FISH has not been performed on domestic cat sperm; therefore, the aim of this study was to visualize sex chromosomes in domestic cat sperm. The results showed the presence of X and Y chromosomes in feline spermatozoa. The procedure used for sperm decondensation and fluorescence in situ hybridization was adequate to visualize chromosomes in domestic cat spermatozoa and, in the future, it may be used to determine the degree of chromosomal abnormalities in these gametes.

## 1. Introduction

Disturbed segregation of chromosomes during gametogenesis can lead to the formation of gametes with an abnormal number of chromosomes. Fertilization with such a gamete may result in the formation of embryos with mutations leading to embryo death or spontaneous abortion [1,2,3]. In addition, the presence of chromosomal abnormalities may have a negative effect on fertility [4]. Fluorescence in situ hybridization (FISH) is a molecular cytogenetics technique that uses fluorescently labeled molecular probes specific to selected chromosomes to enable their visualization in cells. Despite difficulties in carrying out the FISH technique on sperm, related to the need for proper nuclear chromatin decondensation, this technique has been used successfully in the visualization of sperm chromosomes for the determination of structural and numerical chromosomal abnormalities [4,5,6] and nuclear chromatin integrity [7] or the validation of the sperm sexing process [8,9]. Although there are studies in the literature on the use of the FISH technique in humans [10,11], mice [12,13], rats [14], cattle [5,9,15,16], swine [17,18,19,20], horses [4,5,21], and dogs [22,23], FISH has not yet been performed on domestic cat sperm. Therefore, the aim of this study was to visualize the sex chromosomes in domestic cat sperm.

## 2. Material and Methods

According to the Second Local Ethical Committee in Wroclaw, urethral semen collection from patients already sedated for castration procedures did not require agreement of Ethical Committee and this decision had no number.

The frozen semen used in this study came from the Wrocław University of Environmental and Life Sciences, Department of Reproduction and Clinic of Farm Animals semen bank and was shared with another study of domestic cat fertilization. The pooled semen from 4 individuals was thawed (37 °C/30 s) and centrifuged at 3136 rcf/7.5 min, then the supernatant was removed, and the pellet resuspended in 1 mL of Sperm Air medium (Gynemed, Germany). The portion used for this study (20 µL) was then placed in a separate tube, and 1 mL PBS was added. The diluted sperm preparation was stored at −20 °C until analysis.

### 2.1. Decondensation

The thawed sperm suspension (50 µL) was placed in a 1.5 mL Eppendorf tube, and 100 µL of the Buffer A (0.01 M TRIS in 0.9% NaCl) was added. The sample was centrifuged (60 rcf/10 min), the supernatant was withdrawn, and the pellet resuspended in 50 µL of buffer A. The washed sperm suspension (50 µL) was then transferred to a fresh 1.5 mL Eppendorf tube and incubated with 5 µL of 0.02 M dithiothreitol for 1 min, then 5 µL of 0.3 M sodium dodecyl sulphate solution for 10 s, followed by the addition of 50 µL of 100% ethanol. A small drop of the sperm preparation (10 µL) was spotted onto a glass slide and allowed to dry. The preparations were checked for the degree of decondensation of the sperm head using phase-contrast microscopy. The preparations were then dehydrated in 100% ethanol for 5 min and allowed to dry.

### 2.2. Preparation of Molecular Probes

X and Y chromosome-specific painting probes were prepared by laser microdissection (PALM Microlaser system, Carl Zeiss MicroImaging GmbH, Munich, Germany) of the domestic cat chromosomes. DNA amplification was performed using the GenomePlex Single Cell Whole Genome Amplification Kit (Sigma-Aldrich, St. Louis, MO, USA). Chromosome Y was labelled by Orange-dUTPs (Abbott, IL, USA) and chromosome X was labelled by Green-dUTPs (Abbott), using GenomePlex^®^ WGA Reamplification Kit (Sigma-Aldrich) according to the suppliers’ instructions. Prior to use, the probes were denatured at 70 °C for 10 min then put on ice.

### 2.3. Hybridization

Preparations with decondensed domestic cat sperm were digested in pepsin solution for 30 min at 37 °C, before being washed in 2 × SSC (3×/1 min/RT) and then in distilled water for 3 s. The preparations were dehydrated through alcohol series (70%, 90%, and 100%) for 2 min each and then dried (65 °C/30 min). The preparations were denatured in 70% formamide in 2 × SSC for 10 min at 78 °C and immediately translated into 70% ethanol at −20 °C for 4 min, then to 90% and 100% ethanol chilled to −20 °C (4 min each). The slides were dried at room temperature. The previously denatured probes were applied to the slides, covered with a coverslip, and sealed with Fixogum. Hybridization was performed at 37 °C for 1 day.

### 2.4. Posthybridization

After hybridization, the slides were washed in 2 × SSC/RT until the coverslip detached, then rinsed in 50% formamide in 2 × SSC (3 × 5 min/43 °C), followed by 2 × SSC (3 × 5 min/43 °C), before being placed into Detergent Wash Solution (400 mL water + 100 mL 20 × SSC + 250 µL Tween 20) for 10 min at room temperature. The slides were then drained, and 8 µL DAPI (Cambio Biomibo, Warsaw, Poland) was applied before covering with a coverslip.

### 2.5. Microscopic Analysis

Microscopic analysis was performed under a fluorescence microscope (Axio Imager, Zeiss, Jena, Germany) equipped with Zeiss ZEN imager software. Spectrum Green and Spectrum Red filters were used to take the pictures.

## 3. Results

**Figure 1:**
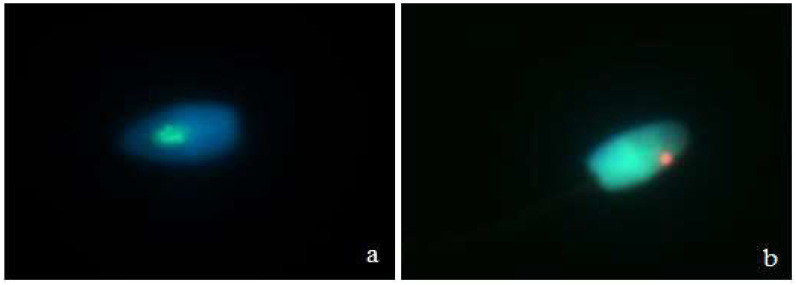
Domestic cat sperm with fluorescent signals specific for the cat’s chromosome X ((**a**), green signal) and Y ((**b**), red signal).

Microscopic analysis showed the presence of X and Y chromosomes in domestic cat spermatozoa (Figure 1). Hybridization efficiency was demonstrated at the level of approximately 90%.

## 4. Discussion

In this study, the sex chromosomes in domestic cat spermatozoa were visualized for the first time following the application of the FISH technique with X and Y whole-chromosome painting probes (WCPP), labeled with fluorochromes. This technique is a useful tool for identifying causes of infertility, including the diagnosis of aneuploidy in sperm [4]. It is believed that chromosomal abnormalities occur more frequently in oocytes than in spermatozoa and arise in the gametogenesis stage as a result of nondisjunction [18,24,25,26]. Nondisjunction may directly influence the occurrence of an unbalanced set of chromosomes in daughter cells and, as a consequence, lead to the death of embryos or to spontaneous abortions [1,3]. Sperm chromosomes have been visualized in humans and many animal species [4,13,14,16,20,23], despite the difficulties associated with the decondensation of nuclear chromatin in the sperm head, which is necessary to obtain a clear and correct result [4]. The FISH technique has been used for many purposes previously, including the determination of the degree of chromosomal abnormalities [4,5,6] and even the validation of the semen sorting procedure [8,9]. Various procedures for decondensation and hybridization of probes with sperm chromatin are described in the literature. In cattle, the decondensation procedure is simpler than in horses [21] and is associated with a higher concentration of disulfide bonds compared to human or mouse sperm [27]. In the case of equine sperm, it is sometimes necessary to adapt the decondensation procedure to the individual characteristics, which further complicates the use of FISH on sperm of this species [21]. In our study, carried out on the pooled semen of various individuals, the hybridization efficiency was approximately 90%. The obtained result suggests that the type of used decondensation is suitable for this species.

In the current study, X and Y chromosome-specific probes were used for hybridization of domestic cat sperm, based on a protocol for decondensation and FISH procedures presented by Bugno-Poniewierska and coworkers [21]. Using these procedures was sufficient to visualize sex chromosomes in feline sperm.

In other animal species to date, signals specific to the X and Y chromosomes and to the EGFR gene in stallions have been visualized [4,21]. A probe for this gene was used for the first time as a hybridization control in ploidy validation studies [4]. For studies on bull sperm, probes specific for X and Y chromosomes [6,7,9,28,29], chromosome 1 [5,17,30], chromosome 6 (locus D6Z1) [7,9], and chromosome 29 have been used [5,30]. Probes for the autosomal 1 and 29 pairs of chromosomes were used to track the segregation of these chromosomes in the gametes of the carriers of these translocations [5,30], while the chromosome 6 probe was used to determine the correlation with perinatal mortality [7]. In boar sperm, probes specific to the X and Y chromosomes [8,20], chromosome 1 [8,18], and chromosome 10 have been used [18]. In the case of canine sperm, probes specific to the X and Y chromosomes [22,23] and to chromosome 1 were used. The probe for chromosome 1 was used as a hybridization control [23].

## 5. Conclusions

The procedure used for sperm decondensation and FISH is adequate to visualize chromosomes in domestic cat sperm and, in the future, it may be used to determine the degree of chromosomal abnormalities in these gametes.

## Data Availability

The data that support the findings of this study are available on request from the corresponding author.
